# Impact of 3D cell culture hydrogels derived from basement membrane extracts or nanofibrillar cellulose on CAR-T cell activation

**DOI:** 10.1016/j.isci.2025.113234

**Published:** 2025-07-30

**Authors:** Sonia Aristin Revilla, Alessandro Cutilli, Eugenia Cambiaso, Dedeke Rockx-Brouwer, Cynthia Lisanne Frederiks, Marc Falandt, Riccardo Levato, Onno Kranenburg, Caroline A. Lindemans, Paul James Coffer, Victor Peperzak, Enric Mocholi, Marta Cuenca

**Affiliations:** 1 Center for Molecular Medicine, University Medical Center Utrecht, Heidelberglaan 100, 3584 CX Utrecht, the Netherlands; 2 Regenerative Medicine Center, University Medical Center Utrecht, Uppsalalaan 8, 3584 CT Utrecht, the Netherlands; 3 Laboratory Translational Oncology, University Medical Center Utrecht, Heidelberglaan 100, 3584 CX Utrecht, the Netherlands; 4 Center for Translational Immunology, University Medical Center Utrecht, Heidelberglaan 100, 3584 CX Utrecht, the Netherlands; 5 Department of Clinical Sciences, Faculty of Veterinary Medicine, Utrecht University, Yalelaan 108, 3584 CM Utrecht, the Netherlands; 6 Department of Orthopaedics, University Medical Center Utrecht, Heidelberglaan 100, 3584 CX Utrecht, the Netherlands; 7 Princess Máxima Center for Pediatric Oncology, Heidelberglaan 25, 3584 CS Utrecht, the Netherlands; 8 Department of Pediatrics, Wilhelmina Children’s Hospital University Medical Center Utrecht, Utrecht, the Netherlands

**Keywords:** Biological sciences, Immune response, Cell biology, Materials science, Biomaterials

## Abstract

Hydrogel-based 3D culture systems are increasingly used for preclinical evaluation of cell-based immunotherapies, including chimeric antigen receptor T (CAR-T) cells. However, hydrogel properties can influence T cell behavior, potentially affecting interpretation of immunotherapy studies. We assessed CD4^+^ T and CAR-T cell responses in two chemically undefined matrices—Matrigel and basement membrane extract (BME)— and in a synthetic nanofibrillar cellulose (NFC) hydrogel. Although NFC was mechanically stiffer, T cell activation and proliferation were higher in NFC than in Matrigel or BME. Murine CD4^+^ T cells acquired a regulatory phenotype in Matrigel and BME but not in NFC. Similarly, CAR-T cell function was reduced in Matrigel and BME but maintained in NFC. These findings underscore how matrix composition can shape T cell responses in 3D culture. NFC provides a chemically defined alternative that preserves T cell activity, supporting its use in more accurate preclinical testing of immunotherapies.

## Highlights

• Basement membrane hydrogels (Matrigel and BME) dampen (CAR-)T cell function
• (CAR-)T cell activity is preserved in synthetic nanofibrillar cellulose (NFC) gels
• T cell proliferation, cytokine secretion are >10-fold lower in Matrigel than in NFC
• CAR-T cell survival and expansion are 10-fold higher in NFC than in Matrigel/BME

## Graphical abstract

**Figure:**
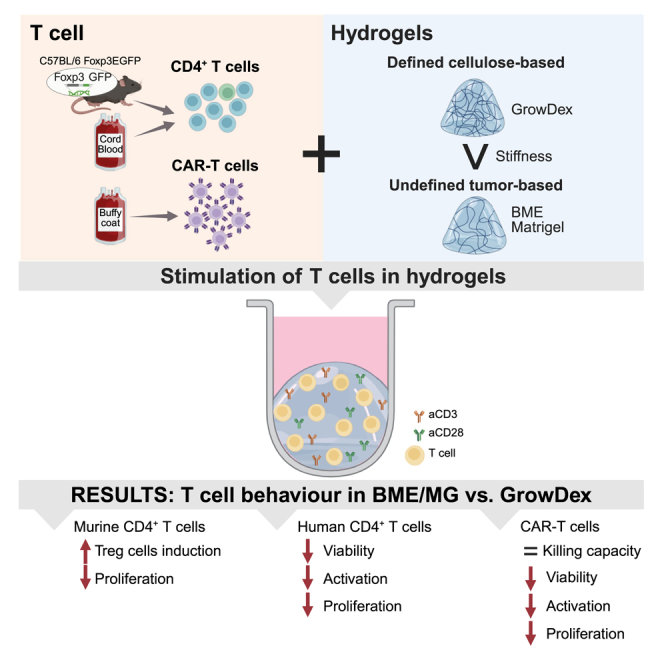

## Introduction

3D cell culture systems hold great potential for advancing biomedical developments in fundamental research, industry, and preclinical testing. *In vitro* 3D models can be generated using healthy or diseased tissues and represent physiologically relevant platforms to study (cancer) cell biology and assess novel drugs, including conventional chemotherapeutics or advanced therapy medicinal products (ATMPs) such as chimeric antigen receptor (CAR)-T cells, tumor-infiltrating lymphocytes (TILs), and bispecific T cell engagers. Over the last decade, significant efforts have been made for developing 3D culture models resembling the tumor microenvironment (TME) that can be used for therapeutic screening, including cytotoxicity assays for testing novel TIL and CAR-T cell products.^1^^,^^2^^,^^3^^,^^4^^,^^5^^,^^6^^,^^7^ The advantages of using 3D culture models for preclinical validation of tumor-targeting T cell products have been increasingly recognized in recent years. In particular, significant investments from both funding and regulatory agencies reflect a growing interest for developing robust, clinically relevant 3D culture systems for testing (engineered) T cell behavior in the context of cancer immunotherapy. Recently, the European Medicine Agency listed the implementation of organoids as tools for preclinical ATMP testing as one of the main proposals to foster ATMP development.^8^

3D cultures rely on matrices made from materials such as hydrogels, polymeric scaffolds, or decellularized extracellular matrix (ECM) components to provide structural support and microenvironmental cues that shape cellular behavior and tissue development.^9^ Thus, matrix choice remains a critical step for developing easy-to-implement and reliable proof-of-concept assays. Importantly, 3D culture-based preclinical drug screenings should be highly reproducible, and it is therefore relevant to base such models on matrices with a defined composition that do not interfere with cell phenotype and activation in an uncontrolled way. Currently, 3D culture models based on animal-derived ECM protein mixtures such as Matrigel or basement membrane extract (BME) are widely used.^10^^,^^11^ Matrigel and BME are obtained from a murine tumor (EHS sarcoma) which must be propagated in mice; at least 25 tumor-bearing mice are needed to prepare a single liter of matrix.^12^ Besides sustainability concerns, a significant limitation of Matrigel and BME is their undefined and variable composition, with unknown amounts of ECM proteins and growth factors.^10^^,^^11^

In the context of 3D (preclinical) tumor-killing assays for evaluating engineered T cell cytotoxicity, the surrounding matrix can influence immune cell phenotype and function, potentially skewing T cell activity. Animal-derived matrices such as Matrigel and BME are inherently heterogeneous and contain diverse ECM components that can be present at variable concentrations. These factors include insulin growth factor-1 (IGF-1), which stimulates T cell growth and differentiation ^13^; transforming growth factor β (TGF-β), which drives the proliferation, differentiation, and expansion of regulatory T (Treg) cells fostering their development toward an immunosuppressive phenotype^14^; and vascular endothelial growth factor (VEGF), which can inhibit T cell antitumor activity and promote Treg cells differentiation.^14^ In addition, it was recently reported that ECM viscoelasticity regulates T cell phenotype —imprinting long-term features— and function, since tuning ECM viscoelasticity results in the generation of functionally distinct T cells.^15^ Thus, to assess T cell function in 3D culture models, it is necessary to use matrices with defined viscoelasticity and devoid of uncontrolled factors that can skew T cell activation and proliferation. This holds particular significance in preclinical ATMP validation, where the ability of a candidate T cell therapeutic product to proliferate and demonstrate anti-tumor activity is critical in determining whether to advance or discontinue further clinical development.

In light of these considerations, the development of next-generation 3D culture systems for assessing T cell function requires a chemically defined hydrogel platform, exempt from undefined components and sourced from non-animal origins. Additional properties that would be desirable in this matrix include commercial availability, ease of implementation and handling (for instance with the possibility to embed cells at room temperature), and compatibility with downstream cellular analysis by flow cytometry, PCR, or RNA sequencing. In this study, we analyze a nanofibrillar cellulose (NFC) hydrogel as a chemically defined alternative to Matrigel or BME with all the above-mentioned features. The NFC fibers self-assemble forming a random 3D structure to support cell growth, differentiation, and spheroid formation.^16^^,^^17^ Here, we show that a culture of murine T cells in Matrigel and BME leads to an increase in the proportion of Treg cells, while this is not observed in NFC. In addition, human CD4^+^ T cells and CAR-T cells exhibit significantly higher activation and proliferation in NFC than in animal-derived matrices, although short-term CAR-T cell cytotoxicity was preserved in all hydrogels. In conclusion, NFC gels provide a chemically defined alternative to Matrigel and BME to preserve (CAR-)T cell effector functions in 3D culture models.

## Results

### NFC hydrogel is stiffer than Matrigel and BME

**Figure 1:**
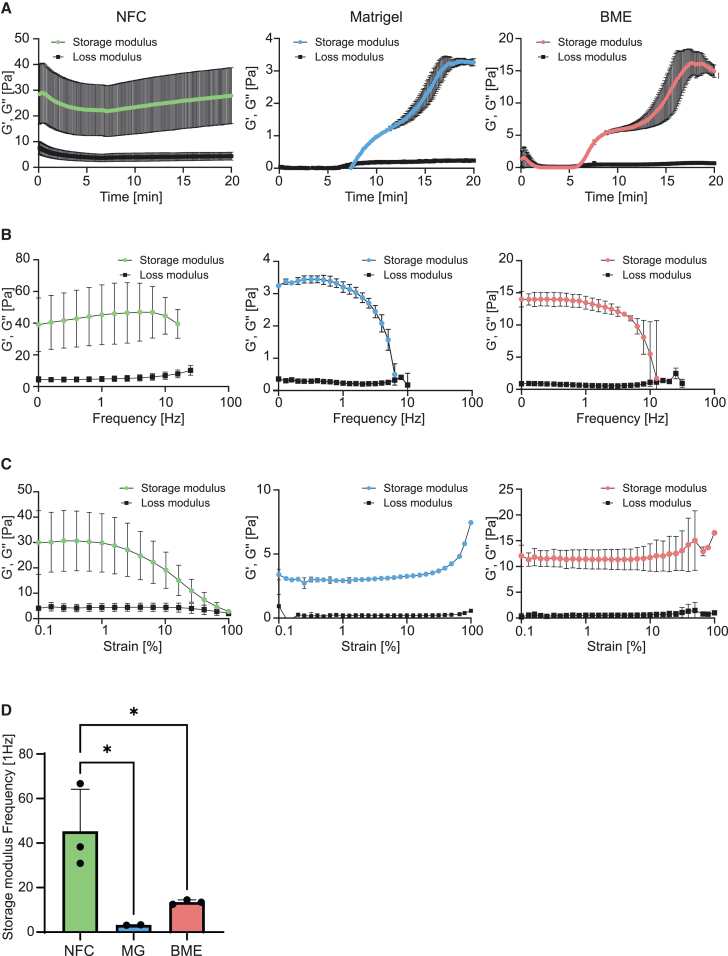
Rheological properties of NFC hydrogel, Matrigel, and BME (A) Rheological time and temperature sweep measurements displaying the crosslinking kinetics and behavior of NFC, Matrigel (MG), and BME, with a temperature ramp from 4°C to 37°C at 5°C per minute before stabilization. (B) Rheological frequency sweep measurements at 37°C, showing the frequency dependent viscoelastic behavior of NFC, Matrigel, and BME. (C) Rheological amplitude sweep at 37°C, showing the amplitude dependent viscoelastic behavior of NFC, Matrigel, and BME. (D) Storage modulus values derived from frequency sweep experiments at a 1 Hz frequency of NFC, MG, and BME. Data representative of three independent measurements are presented as means ± SD. Data were analyzed using unpaired one-way ANOVA with Tukey’s multiple comparison test (∗*p* < 0.05). NFC, nanofibrillar cellulose hydrogel; MG, Matrigel; BME, basement membrane extract.

First, the viscoelastic behavior of NFC hydrogels, Matrigel, and BME was assessed using oscillatory rheological analysis. A time/temperature ramp from 4°C to 37°C at 5°C per minute before stabilization was used to analyze the crosslinking kinetics and stiffness of the viscoelastic hydrogel materials, since both Matrigel and BME display a temperature-dependent gelation mechanism (Figure 1A). Matrigel and BME displayed similar crosslinking kinetics. Our results showed a higher stiffness of NFC hydrogel when compared to Matrigel or BME. The storage modulus of NFC remained stable across the analyzed temperature range (Figure 1A), indicating that in contrast to Matrigel and BME, changes in temperature have only a minor effect on the viscoelastic properties of NFC hydrogels. As previously reported,^18^ the rheology of the NFC hydrogels shows reversible gelation: at high stress levels (e.g., injections or pipetting) a fluid-like behavior is observed whereas at low stress level and quiescent conditions a stepwise transition to solid-like behavior is observed. In addition, high viscosity (i.e., 3D gel structure) is restored within a few seconds after shearing (injection or mixing) stops. This practically means that the cells can be encapsulated inside NFC gels over a broad temperature range, as NFC will behave as a fluid while it is being pipetted and will regain a 3D, solid-like structure immediately after mixing. In contrast, Matrigel and BME need to be manipulated at cold temperatures when encapsulating cells not to trigger unwanted thermal crosslinking too early.

Next, a frequency sweep was applied to the materials to study the time-dependent behavior of the hydrogels in response to a non-destructive deformation (Figure 1B). These results showed that especially NFC seems to be more resilient to faster deformations than Matrigel and BME, while Matrigel showed the least resilience to fast deformations. To further characterize the hydrogels, an amplitude sweep was performed to assess the range of strain the materials can withstand before yielding (Figure 1C). Both Matrigel and BME showed a strain stiffening behavior when subjected to shear strain values > 50%. Conversely, in line with the particulate nature of the hydrogel, NFC displayed a reduction in storage modulus with increasing shear amplitude, suggesting that elevated strains promote a more fluid-like behavior of the gel by disrupting the interactions that keep the NFC slurry cohesive. This aspect can be beneficial to mechanically disaggregate the gel to retrieve embedded cells. Finally, to provide a comparison of the stiffness values of the different hydrogels, we extracted the storage moduli of the materials at 1 Hz (Figure 1D). The values derived from the frequency sweep assays demonstrate that NFC is significantly stiffer than both Matrigel and BME, although all gels present values comparable to soft tissues, in the range between 3 and 40 Pa of shear storage moduli, typically suitable for cell culture and to perform migration and invasion assays.

### Culture of murine CD4^+^ T cells in Matrigel and BME leads to an increase in Treg cell numbers, while this is not observed in NFC hydrogels

**Figure 2:**
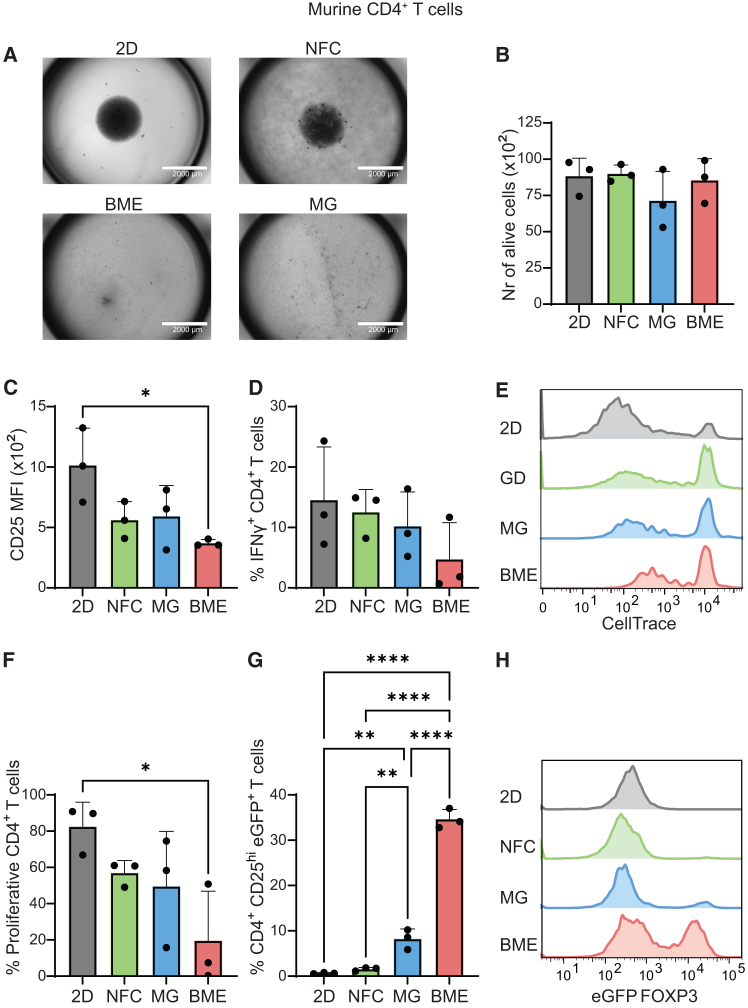
ECM-derived hydrogels (Matrigel and BME) influence murine CD4^+^ T cell activation, function and differentiation to regulatory T cells (A) Representative bright field images (EVOS microscope, scale bar 2000 μm) of CD4^+^ T cells purified from Foxp3eGFP mice stimulated *ex vivo* with anti-CD3 (1 μg/mL) and anti-CD28 (1 μg/mL) in standard 2D suspension (control) or embedded in different hydrogels (NFC, Matrigel, and BME). CD4^+^ T cells were analyzed by flow cytometry after 5 days in culture. (B) Number of viable CD4^+^ T cells recovered after culture. (C) Median fluorescent intensity (MFI) of CD25 in CD4^+^ T cells. (D) Percentage of IFNγ-expressing CD4^+^ T cells. (E and F) Representative histograms showing CellTrace Violet expression on CD4^+^ T cells (E) and proportion of proliferative CD4^+^ T cells (f; CTV-low). (G and H) Percentage of Foxp3 eGFP^+^ CD25^hi^ Treg cells (G) and representative histograms showing Foxp3 expression on Foxp3-eGFP^+^ Treg cells (H). Each point represents an individual mouse. Data from three independent experiments are presented as means ± SD. Data were analyzed using unpaired one-way ANOVA with Tukey’s multiple comparison test (∗*p* < 0.05, ∗∗*p* < 0.005, ∗∗∗∗*p* < 0.0001). NFC, nanofibrillar cellulose; MG, Matrigel; BME, basement membrane extract.

We next explored how NFC, Matrigel, and BME impact the activation of embedded murine CD4^+^ T cells isolated from transgenic C57BL/6 Foxp3eGFP mice. In this mouse model, eGFP expression is controlled by the promoter of *Foxp3*, the key transcription factor driving Treg cell differentiation and function.^19^^,^^20^ Consequently, eGFP serves as a surrogate marker for the identification of Treg cells. T cell activation involves a three-step process: first, antigen recognition through the TCR; second, CD28 co-stimulation; and, finally, cytokine-mediated triggering of interleukin-2 receptor (IL-2R or CD25) signaling. In our experimental set-up, T cells were stimulated *in vitro* using anti-CD3/CD28 monoclonal antibodies along with soluble IL-2. T cell activation induces changes in their morphology with an increase in size and formation of clusters, which are visible under the microscope. Notably, after five days in culture, CD4^+^ T cells cultured in 2D and in NFC exhibited these characteristic clusters, while this was not observed for CD4^+^ T cells embedded in Matrigel and BME (Figure 2A). Next, CD4^+^ T cell viability in the different gels was evaluated by flow cytometry. We found that all conditions yielded a similar number of viable cells (Figure 2B). We then examined additional parameters related to CD4^+^ T cell activation such as CD25 expression, which is upregulated in activated T cells. We observed that CD25 expression was reduced when CD4^+^ T cells were cultured in BME (MFI 370 ± 24) as compared to NFC (560 ± 125) or Matrigel (590 ± 207), suggesting reduced T cell activation (Figure 2C).

To further investigate functional aspects of T cell activation, we assessed the fraction of CD4^+^ T cells expressing interferon gamma (IFNγ) using flow cytometry. Our analysis showed similar proportions of IFNγ^+^ CD4^+^ T cells among the different culture conditions (Figure 2D), suggesting that the functional capacity of CD4^+^ T cells to produce this cytokine is preserved independently of the matrix used, although there was a non-significant trend toward decreased IFNγ production in BME. In parallel, CD4^+^ T cell proliferation was evaluated by measuring CellTrace Violet dilution by flow cytometry. Proliferation of CD4^+^ T cells embedded in BME was significantly decreased (19.5% ± 22% proliferative cells) compared to proliferation in 2D (82.5% ± 11%) and lower than in NFC (57% ± 6%) and Matrigel (49.5% ± 25%; Figures 2E–2F). Finally, we analyzed the proportion of CD4^+^ T cells expressing Foxp3 eGFP within our cultures to assess changes in Treg numbers in the different matrices. A significant increase in the percentage of Treg cells (defined as Foxp3 eGFP^+^ CD25hi CD4^+^ T cells) was observed when activated CD4^+^ T cells were embedded in Matrigel (8.2% ± 1.8%) or BME (34.6% ± 1.8%) for five days. In contrast, embedding activated CD4^+^ T cells in NFC did not increase Treg cell numbers (Figures 2G, 2H, and S1). Taken together, these data highlight that ECM-derived matrices such as Matrigel and BME can affect murine CD4^+^ T cell activation, proliferation and differentiation toward a regulatory phenotype, while this is not observed in NFC gels.

### Increased human CD4^+^ T cell viability, activation, and proliferation in NFC compared to ECM-derived hydrogels

**Figure 3:**
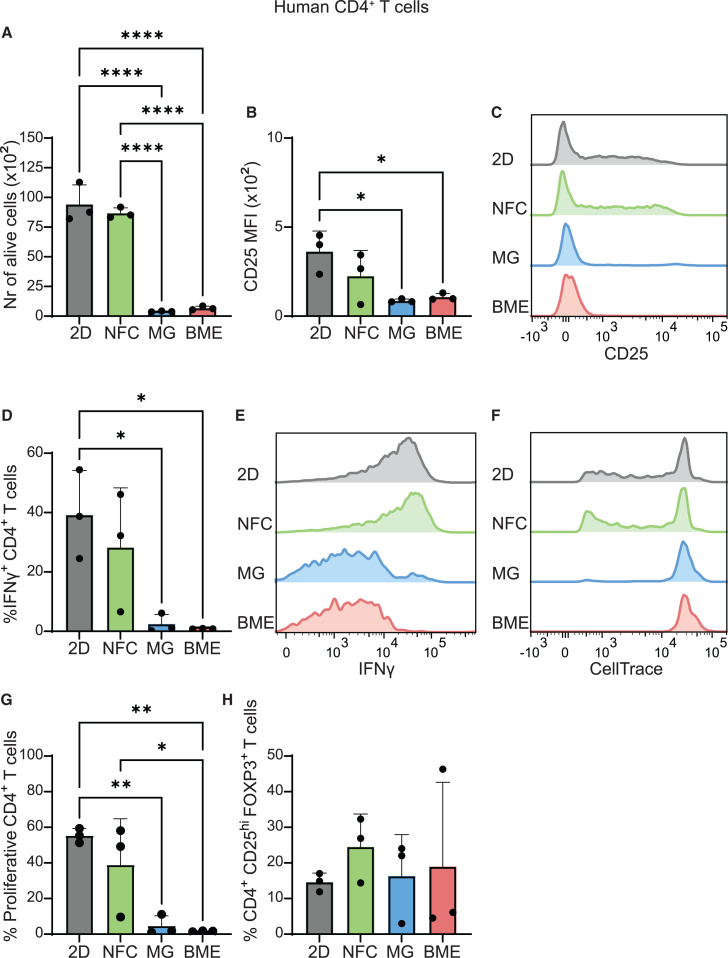
ECM-derived hydrogels (Matrigel and BME) hamper human CD4^+^ T cell activation and function (A–H) CD4^+^ T cells isolated from cord blood mononuclear cells were stimulated *ex vivo* with anti-CD3 (1 μg/mL) and anti-CD28 (1 μg/mL) for 5 days in standard 2D suspension (control) or embedded in NFC, Matrigel and BME. (A) Number of viable CD4^+^ T cells recovered after culture. (B and C) Median fluorescent intensity (MFI) of CD25 in CD4^+^ T cells (B) and representative histograms showing CD25 expression on CD4^+^ T cells (C). (D and E) Percentage of IFNγ-expressing CD4^+^ T cells (D) and representative histograms showing IFNγ expression on CD4^+^ T cells (E). (F and G) Representative histograms showing CellTrace Violet expression on CD4^+^ T cells (F) and proportion of proliferative CD4^+^ T cells (CTV-low; G). (H) Percentage of FOXP3^+^ CD25^hi^ Treg cells. Each point represents an individual donor. Data from three independent experiments are presented as means ± SD. Data were analyzed using unpaired one-way ANOVA with Tukey’s multiple comparison test (∗*p* < 0.05, ∗∗*p* < 0.005, ∗∗∗∗*p* < 0.0001). NFC, nanofibrillar cellulose; MG, Matrigel; BME, basement membrane extract.

Next, we explored how NFC, Matrigel, and BME influence the phenotype and function of human CD4^+^ T cells. We examined viability, activation markers, proliferation, and spontaneous Treg induction in the different hydrogels after five days in culture. CD4^+^ T cells were isolated from cord blood of human donors and stimulated *in vitro* using anti-CD3/CD28 monoclonal antibodies along with soluble IL-2, following the same protocol as for murine T cells. CD4^+^ T cell viability was significantly higher in NFC than in Matrigel and BME (Figure 3A). T cell activation, as measured by CD25 expression, was lower in Matrigel (MFI 87 ± 7.7) and BME (107 ± 16) than in NFC (225 ± 117) and 2D controls (363 ± 93) (Figures 3B and 3C). To functionally evaluate activated CD4^+^ T cells in the different matrices, the percentage of CD4^+^ T cells expressing IFNγ was measured by flow cytometry. CD4^+^ T cells cultured in NFC produced more IFNγ (28.3% ± 16% IFNγ^+^ cells) than cells cultured in Matrigel (2.5% ± 2.6%) and BME (0.9% ± 0.1%) (Figures 3D, 3E, and S2). In addition, CD4^+^ T cell proliferation in NFC was also significantly higher (39% ± 21% proliferative cells) compared to BME (1.6% ± 0.19%) and was also higher than in Matrigel (4.7% ± 4.5%) (Figures 3F and 3G). In contrast to murine CD4^+^ T cells, we found that neither Matrigel nor BME promoted an increase in Treg cell numbers (Figure 3H). Taken together, our findings demonstrate that survival and activation of human CD4^+^ T cells is higher in synthetic NFC gels than in ECM-derived matrices.

### CAR-T cell cytotoxicity in short term cultures is comparable between NFC, Matrigel, and BME

**Figure 4:**
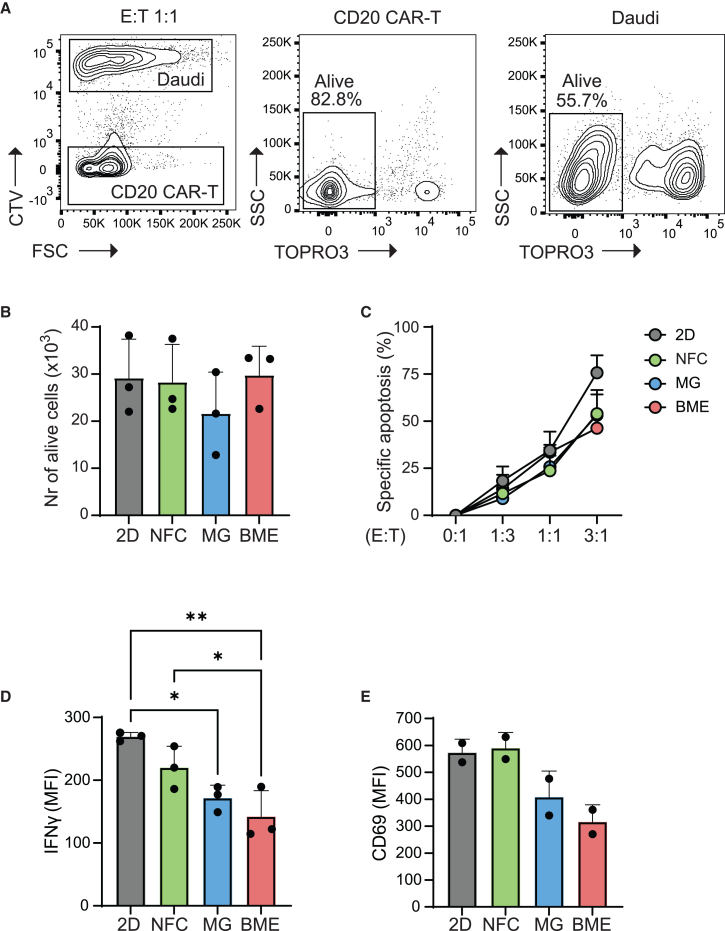
CAR-T cell cytotoxicity in short term cultures is comparable between NFC, Matrigel, and BME CD20 CAR-T cells were co-cultured with Daudi (Burkitt lymphoma cell line) cells labeled with CellTrace Violet (CTV) for 24 h at the different effector to target (E:T) ratios specified, in standard 2D suspension (control) or embedded in the specified hydrogels. (A) Representative gating strategy for analyzing the viability of Daudi and CD20 CAR-T cells by flow cytometry. (B) Number of viable CAR-T cells recovered after culture in the 1:1 E:T condition (50,000 Daudi +50,000 CAR-T cells/well) as measured by flow cytometry using counting beads (Flow-Count fluorospheres). Data were analyzed using a one-way ANOVA with Tukey’s multiple comparison test. (C) Specific lysis (%) of Daudi cells induced by CD20 CAR-T cells at the specified E:T ratios. Specific apoptosis was calculated by applying the following formula: [(%viable untreated − %viable treated)/%viable untreated] × 100. (D and E) Bar plot showing median fluorescence intensity (MFI) of (D) IFNγ and (E) CD69 in CAR-T cells cultured for 24 h in the described conditions. Data from two or three independent experiments are presented as means ± SD. Data were analyzed using a two-way ANOVA with Tukey’s multiple comparison test (∗*p* < 0.05, ∗∗*p* < 0.005). NFC, nanofibrillar cellulose; MG, Matrigel; BME, basement membrane extract.

CAR-T cells are engineered immune cells designed to identify and eradicate cancer cells by recognizing distinct tumor-associated antigens. *In vitro* assays testing CAR-T cell cytotoxicity against cancer cells are critical for understanding the biology of the interactions between CAR-T and tumor cells, and for screening new drug candidates. Here, we evaluated how NFC, Matrigel, and BME impact the cytotoxicity of CD20 CAR-T cells against Daudi cells, a Burkitt’s lymphoma cell line. In our experiments, CD20 CAR-T cells were co-cultured with Daudi cells at various effector-to-target ratios (E:T) in each hydrogel. After 24 h of co-culture, the viability of CD20 CAR-T and Daudi cells was analyzed by flow cytometry. A comparable number of viable CD20 CAR-T cells was recovered across the different culture conditions (Figures 4A and 4B). Regarding CAR-T cytotoxicity, a dose-dependent correlation between increasing E:T ratios and target cell apoptosis could be observed in all matrices. CAR-T mediated killing was comparable in the three hydrogels (Figure 4C). While cytotoxicity against target cells was similar across gels, CAR-T cells embedded in Matrigel and BME secreted a significantly reduced amount of IFNγ (measured by FACS) as compared to 2D and NFC cultures [IFNγ MFI 24 h: 171 ± 11.9 (Matrigel) and 142 ± 23.8 (BME) vs. 269 ± 3.9 (2D) and 220 ± 19.6 (NFC)]. Expression of the early T cell activation marker CD69 was also lower in Matrigel and BME than in 2D or NFC [CD69 MFI 24 h: 408 ± 68.3 (Matrigel) and 315 ± 45.3 (BME) vs. 573 ± 35.5 (2D) and 590 ± 41.3 (NFC)] (Figures 4D and 4E). Overall, these findings indicate that CAR-T mediated cytotoxicity in short-term (24 h) co-cultures is preserved in NFC, Matrigel, and BME.

### CAR-T cell activation and proliferation is preserved after a 4- or 10-day stimulation in NFC, but not in Matrigel or BME

**Figure 5:**
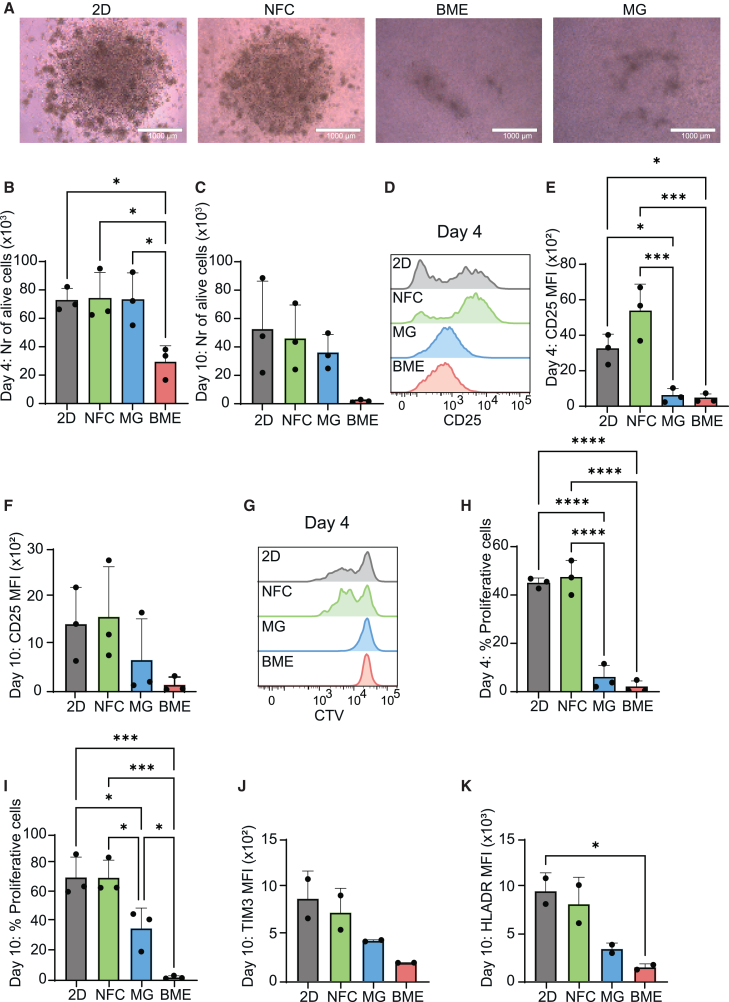
ECM hydrogels (Matrigel and BME) reduce CAR-T cell activation and proliferation (A) Representative bright field images (scale bar, 1,000 μm) of CD20 CAR-T cells stimulated *in vitro* for 4 days with coated anti-CD3 (1.6 μg/mL) and soluble anti-CD28 (1 μg/mL) in either standard 2D suspension (control) or the indicated gels. (B and C) Number of viable CAR-T cells recovered after 4 (B) or 10 days (C) of culture as measured by flow cytometry using counting beads (Flow-Count fluorospheres). (D) Representative histograms showing CD25 expression in CAR-T cells. (E and F) median fluorescence intensity (MFI) of CD25 in CAR-T cells after 4 (E) or 10 days (F) of culture. (G–I) For flow cytometry-based analysis of proliferation, CD20 CAR-T cells were labeled with CellTrace Violet (CTV) dye on day 0 and stimulated as indicated in (A). (G) Representative histograms showing CTV expression on CAR-T cells and (H and I) proportion of proliferative CAR-T cells (low CTV expression) after (H) 4 or (I) 10 days of culture. (J and K) Median fluorescence intensity (MFI) of (E) TIM3 and (F) HLADR in CAR-T cells after 10 days culture. Data from two or three independent experiments are presented as means ± SD. Data were analyzed using unpaired one-way ANOVA with Tukey’s multiple comparison test (∗*p* < 0.05, ∗∗∗*p* < 0.0005, ∗∗∗∗*p* < 0.0001). NFC, nanofibrillar cellulose; MG, Matrigel; BME, basement membrane extract.

Since preclinical CAR-T cell testing often involves culture periods longer than 24 h, we evaluated how the activation and functionality of CD20 CAR-T cells evolves over longer-term cultures in different 3D matrices. To address this, we cultured CD20 CAR-T cells in NFC, Matrigel, and BME for either 4 or 10 days in the presence of IL-2, anti-CD3 and anti-CD28 monoclonal antibodies to induce proliferation. Morphological changes associated with cell activation, such as cell enlargement and the formation of distinctive clusters, were particularly pronounced in CAR-T cells cultured in NFC (Figure 5A). The number of alive CD20 CAR-T cells retrieved from the different culture conditions was quantified by flow cytometry. CAR-T cell viability after a 4-day culture was significantly reduced in BME (27,706 ± 11,315 alive cells) as compared to NFC (72,836 ± 18,082), 2D or Matrigel (Figure 5B), and the same trend was observed after 10 days in culture (Figure 5C). In line with our previous findings, CD25 expression was significantly lower when CAR-T cells were cultured for 4 days in Matrigel (MFI 500 ± 401) and BME (407 ± 251) compared to NFC (5,194 ± 1,528; Figures 5D and 5E), and differences were maintained after 10 days in culture (Figure 5F). In addition, CAR-T cells embedded in NFC displayed higher proliferation rates after 4 days in culture (46.8% ± 7.2% proliferative cells) than in Matrigel (4.5 ± 5.1) or BME (0.9 ± 2.6) (Figures 5G and 5H), and these statistically significant differences were maintained after 10 days in culture (Figure 5I). Expression of the late T cell activation markers TIM-3 and HLA-DR after a 10-day culture was lower in Matrigel and BME than in 2D or NFC (Figures 5J and 5K). Thus, our data indicates that over longer culture periods, ECM-derived hydrogels can negatively impact CAR-T cell activation and proliferation.

## Discussion

3D culture systems are more versatile than 2D monolayer cultures, offering the possibility to mimic more accurately different cellular environments.^15^^,^^21^^,^^22^^,^^23^^,^^24^ Still, establishing 3D culture platforms that are at the same time reproducible, high-throughput and compatible with both tumor and immune cell functions remains a challenge for the development of (personalized) models for testing immunotherapeutic drugs. It is important to ensure that the chosen matrix does not interfere with immune cell function in an uncontrolled manner. In this study, we compare (CAR-)T cell activation and proliferation in Matrigel, BME, and NFC, a nanocellulose-based synthetic hydrogel. Despite the effectiveness of Matrigel and BME in culturing several types of organoids, animal-derived matrices are limited by their non-defined composition, with varying amounts of ECM components and growth factors leading to reproducibility issues.^10^^,^^11^^,^^25^^,^^26^ Here, we explore NFC as a chemically defined alternative and propose it as a solution to prevent uncontrolled skewing of T cell phenotype in 3D cultures.

T cell phenotype *in vivo* is influenced by the anatomical location, motivating recent studies to optimize culture conditions and matrix composition for investigating T cell biology *in vitro.*^15^^,^^21^^,^^23^^,^^24^^,^^27^^,^^28^ Previous reports have explored the suitability of chemically and non-chemically defined matrices as 3D scaffolds for culturing T cells, showing superior T cell expansion in 3D as compared to standard 2D culture.^24^ Importantly, the mechanical properties of hydrogels, such as stiffness and viscoelasticity, affect T cell phenotype and activation.^15^^,^^23^^,^^24^^,^^29^^,^^30^ Considering the structural properties and the biochemical composition of the three hydrogels analyzed, and building on data from previous studies, superior T cell activation in NFC as compared to Matrigel/BME is likely achieved by a combination of its rheological properties (i.e., higher stiffness) and the absence of inhibitory T cell factors that can be present in undefined gels. Here we show that NFC is significantly stiffer (higher storage modulus, *G′*) than Matrigel or BME (Figure 1). Our data are in line with previous reports characterizing NFC hydrogel rheology.^18^ Stiffer 3D environments generally lead to increased CD4^+^ T cell activation and expansion.^24^^,^^29^^,^^30^ Thus, different studies have shown that T cell migration and secretion of IL-2, IL-12, IL-22, and IFNγ is higher in stiffer gels,^31^^,^^32^^,^^33^ and revealed that this stiffness-mediated activation of lymphocytes is strongly associated with CD3 signaling.^29^^,^^34^ Matrix stiffness modulates TCR mechanotransduction, regulating T cell activation and proliferation.^23^^,^^24^^,^^29^^,^^30^ In addition, pore size of NFC hydrogels (13 μm in average) ^35^ is substantially larger than that of Matrigel (140–350 nm).^36^ This allows better diffusion and consequently higher availability of stimuli such as anti-CD3 and anti-CD28 antibodies, which likely contributes to the increased T cell activation observed in NFC hydrogels. Importantly, we report for the first-time induction of murine Treg cells when CD4^+^ T cells were embedded within Matrigel and BME, while this was not observed in NFC (Figures 2G and 2H). This finding aligns with a previous study where murine CD4^+^ T cell activation was analyzed in PDMS substrates with variable stiffness, showing a significant increase in Treg cell numbers on lower stiffness substrates.^37^ In addition, our data showed enhanced viability, activation, and expansion of human CD4^+^ T cells embedded in NFC compared to Matrigel and BME (Figures 3A–3G), supporting the notion that stiffer hydrogels favor T cell activation. In contrast to murine T cells, we did not observe Treg induction from naive human CD4^+^ T cells in Matrigel or BME (Figure 3H). This discrepancy could be explained by species-specific responses to matrix composition. Matrigel and BME are BMEs obtained from a mouse sarcoma, and therefore it is possible that murine CD4^+^ T cells are more sensitive to the biochemical cues present in these gels than human T cells. Matrigel and BME contain transforming growth factor (TGF)-β and VEGF, which can induce murine Treg cells.^11^^,^^14^^,^^25^ Moreover, both TGF-β and VEGF can inhibit CD4^+^ T cell activation,^11^^,^^14^^,^^25^ which could underlie the observed absence of activation in human CD4^+^ T cells within Matrigel and BME matrices. Taken together, our data highlights the need for considering both matrix composition and stiffness when establishing 3D cell culture systems for analyzing T cell biology.

This is the first study addressing how CAR-T cell performance can be influenced by the 3D culture matrix. (Immuno)therapy testing in high-throughput, reproducible 3D models for solid and hematologic malignancies is increasingly becoming an essential step in preclinical development.^38^^,^^39^ CAR-T cell products undergo a rigorous functional evaluation before entering clinical development. Assays for preclinical CAR-T cell testing include tumor killing assays (in 2D plates and organoids), evaluation of proliferation and cytokine secretion, and phenotypical analyses of activation, memory features and exhaustion.^40^ Matrigel and BME are widely used as hydrogel scaffold for organoid research,^1^^,^^41^ including 3D models for assessing CAR-T or TIL cytotoxicity.^42^^,^^43^^,^^44^^,^^45^^,^^46^ Here, we show that CAR-T cell activation and proliferation after anti-CD3 stimulation can be severely impaired in Matrigel and BME, and this reduction was not observed when CAR-T cells were cultured in NFC (Figure 5). Thus, Matrigel and BME can dampen CAR-T cell activation and proliferation in an uncontrolled manner, which could lead to underestimating the efficacy of potentially relevant CAR-T products at the preclinical stage. In contrast, CAR-T cell activation, proliferation, cytokine secretion and cytotoxicity are preserved in NFC hydrogel, making it an attractive candidate for preclinical CAR-T cell testing. A potential challenge is that the suitability of NFC as scaffold for some types of organoids remains to be characterized. Of note, CAR-T cell cytotoxicity was comparable across all hydrogels (Figure 4), suggesting that while Matrigel and BME may hinder the downstream TCR signaling events leading to T cell expansion, CAR-mediated recognition is preserved. In the context of tumor immunology, previous reports have shown upregulation of the inhibitory receptors PD-1 and CTLA-4 in CD4^+^ TILs cultured with Matrigel-inlaid melanoma organoids.^43^ In an *in vivo* mouse model, co-implantation of the breast cancer cell line 4T1 with Matrigel, as opposed to implantation in PBS, was reported to impair T cell migration, activation, and function within the xenograft tumors.^47^ Matrigel reduced *in vivo* T cell infiltration in the tumors, decreased the percentage of IFNγ secreting T cells, and abolished T cell activation as measured by CD69 and CD44 expression.^47^ This study showed that laminin-111, one of the most abundant matrisome proteins in Matrigel,^48^ significantly inhibits T cell activation and proliferation.^47^ Of note, in most mature tissues, laminin-111 is not as abundant as in Matrigel.^49^ Matrigel-induced immunosuppression is also driven by the presence of collagen. Collagen type IV represents ∼30% of the total Matrigel composition.^50^ Multiple collagen types, including collagen type I, IV, and VI, can drive T cell exhaustion by binding the leukocyte-specific collagen receptor LAIR1 and impairing T cell activation.^51^ Thus, CAR-T cell activation in Matrigel can be skewed by the presence of different combinations of collagen subtypes in variable amounts. Of note, the ECM is dynamic and tissues have unique ECM compositions adapted to their needs, which can be further modified in pathological conditions.^52^ Collagen and laminin gene expression profiles are highly tissue- and tumor-specific.^48^^,^^53^ Therefore, despite being rich in collagen and laminin, the mouse sarcoma-derived ECM present in Matrigel and BME does not provide a representative niche for either tumors or healthy tissues. In contrast, NFC gels can be supplemented on demand with relevant ECM components to recreate tissue-specific matrices.^54^ Importantly, CAR-T cell performance is also influenced by their intrinsic phenotype and differentiation stage. Successful clinical response to CAR-T cell therapy has been associated to CAR-T upregulation of genes related to a memory cell phenotype.^55^ Previous reports show that after a 5-day culture, T cells embedded in Matrigel express significantly lower levels of the memory marker CD45RO than cells cultured in 2D or a 3D polystyrene scaffold.^24^ Together with our observations of reduced CAR-T cell proliferation and CD25 expression in Matrigel and BME as compared to NFC, these data highlight the relevance of using chemically defined hydrogels for testing T cell tumor reactivity.

In summary, we report that the intrinsic cytotoxic and proliferative potential of (CAR-)T cells can be underestimated when performing assays in 3D cultures based on Matrigel or BME. As an alternative, we suggest the use of chemically defined synthetic gels, and we show that NFC hydrogels are suitable 3D matrices for preserving T cell phenotype and activation.

### Limitations of the study

This work describes how T cell activation and proliferation can be significantly impaired in 3D cultures using BME or Matrigel, and presents NFC hydrogels as an alternative to preserve T cell function in preclinical evaluation of novel immunotherapy candidates. Considering that a growing number of preclinical assays rely on the use of organoids, the suitability of NFC as a platform for organoid culture should be further explored. In addition, this study was limited to the analysis of CD20 CAR-T cell cytotoxicity against a lymphoma cell line. The influence of hydrogel composition in the function of CAR-T cells targeting other tumor types must be characterized in future studies.

## Resource availability

### Lead contact

Further information and requests for resources or reagents should be directed to and will be made available upon reasonable request by the lead contact: Marta Cuenca (m.cuenca@umcutrecht.nl).

### Materials availability

This study did not generate new unique reagents.

### Data and code availability

• Data: All data supporting the findings of this study are available from the lead contact upon reasonable request.
• Code: This paper does not report original code.
• Additional information: Any additional information required to reanalyze the data reported in this paper is available from the lead contact upon request.

## Acknowledgments

We thank Ralph G. Tieland for assistance with CAR-T cell cultures. We would like to thank Suze Jansen, Angelo Meringa, and all members of the Coffer, Peperzak, Levato, Kranenburg and Lindemans group for helpful discussions. We thank the FACS facilities of the University Medical Center Utrecht (UMCU) and the Hubrecht Institute for their support. We would like to express our gratitude to all employees of NFCL Utrecht for their assistance with mice experiments. Funding: This work was supported in part by a Worldwide Cancer Research grant (ref. 19-0371) and Marie S. Curie Co-fund RESCUE grant (ref. 801540). The funding agencies played no role in the design, reviewing, or writing of the manuscript.

## Author contributions

S.A.R.: Methodology, formal analysis, investigation, writing – original draft, review and editing, and visualization; A.C.: Methodology, formal analysis, and investigation; E.C.: Methodology, formal analysis, and investigation. D.R.-B.: Investigation; C.L.F.: Investigation; M.F.: Methodology, formal analysis, investigation, and visualization; R.L.: writing – review and editing and supervision; O.K.: Writing – review and editing and supervision; C.A.L.: Supervision; P.J.C.: Writing – review and editing, supervision, and funding acquisition; V.P.: Writing – review and editing and supervision; E.M.: Conceptualization, methodology, writing – review and editing, supervision, and project administration; M.C.: Conceptualization, methodology, formal analysis, investigation, writing – original draft, review and editing, visualization, supervision, and project administration.

## Declaration of interests

The authors declare no conflicts of interest.

## STAR★Methods

### Key resources table

**Table:** 

REAGENT or RESOURCE	SOURCE	IDENTIFIER
**Antibodies**		

Anti-Mouse CD3e functional grade	eBioscience	Cat# 16-0031-85; RRID: AB_468847
Anti-Mouse CD28 functional grade	eBioscience	Cat# 16-0281-81; RRID: AB_468924
Anti-Human CD3e functional grade	eBioscience	Cat# 16-0037-81; RRID: AB_468854
Anti-Human CD28 functional grade	eBioscience	Cat# 16-0289-85; RRID: AB_468926
Anti-Mouse CD4 APC	Biolegend	Cat# 100516; RRID: AB_312718
Anti-Mouse CD25 Pacific Blue	Biolegend	Cat# 102022; RRID: AB_493642
Anti-Mouse IFNg PECy7	Biolegend	Cat# 505826; RRID: AB_2295770
Anti-Human CD4 FITC	Biolegend	Cat# 300506; RRID: AB_2562052
Anti-Human CD69 FITC	Miltenyi	Cat# 130-113-523; RRID: AB_2733656
Anti-Human CD4 PECy7	eBioscience	Cat# 25-0049-42; RRID: AB_1659695
Anti-Human CD25 AF488	eBioscience	Cat# 53-0259-42; RRID: AB_2043827
Anti-Human TIM3 PE	Biolegend	Cat# 345006; RRID: AB_2116576
Anti-Human HLA-DR PECy7	Biolegend	Cat# 307616; RRID: AB_493588
Anti-Human CD25 APC	Biolegend	Cat# 302610; RRID: AB_314279
Anti-Human FOXP3 PE	Biolegend	Cat# 320108; RRID: AB_492986
Anti-Human IFNg PECy7	BD Biosciences	Cat# 557643; RRID: AB_396760

**Chemicals, peptides, and recombinant proteins**		

PBS (1X) without Ca++, Mg++, 500ml	Lonza	BE17-516F
FBS	Gemini Bio-Products	100-106
RPM1 1640 medium with Glutamax	Gibco	11534526
Penicillin- Streptomycin	Thermo Fisher Scientific	15070063
Sodium pyruvate	Gibco	11360070
1X nonessential amino acids	Gibco	11140050
2-mercaptoethanol	Sigma-Aldrich	M6250
1M HEPES buffer	Gibco	15630080
Recombinant murine FGF-basic	PeproTech	450-33
*N*-acetylcysteine	Sigma	A9165-5g
B-27™ Supplement(50X), serum-free	Gibco	17504044
Cultrex RGF BME, Type 2	R&D systems	3533-005-02
Matrigel, GFR	Corning	CLS356231-1EA
GrowDex	UPM Biomedicals	100.103.905
GrowDase	UPM Biomedicals	100.103.905
Cell Recovery Solution	Corning	CLS354270
EDTA	Sigma-Aldrich	E6511
Trypan Blue	Sigma-Aldrich	72-57-1
Recombinant Human IL-2 Protein	PeproTech	200-02
Recombinant Human TGF-beta 1	R&D systems	7754-BH-025
Ficoll Paque Plus	GE Life Sciences	17144003
GolgiStop	BD Biosciences	554724
Fugene HD	Promega	E2312
Polybrene	Merck	TR-1003-G

**Critical commercial assays**		

CD4 (L3T4) MicroBeads, mouse	Miltenyi Biotec	130-117-043
LS Columns	Miltenyi Biotec	130-042-401
QuadroMACS® MultiStand	Miltenyi Biotec	130-042-303
MagniSort human CD4^+^ T cell enrichment kit	Thermo Fisher Scientific	8804-6811-74
BD IMag Cell Separation Magnet	BD Biosciences	552311
Foxp3/Transcription Factor Staining Buffer Set	eBioscience	#00-5523-00
Zombie NIR™ Fixable Viability Kit	Biolegend	423106
CellTrace™ Violet Cell Proliferation Kit	Invitrogen	C34557
TO-PRO-3 iodide (642/661)	Thermo	10710194
Dynabeads™ Human T-Activator CD3/CD28	Gibco	11161D
Flow count Fluorospheres	Beckman Coulter	7547053

**Experimental models: Cell lines**		

Daudi	DSMZ	ACC 78
Phoenix-AMPHO	ATCC	CRL-3213

**Experimental models: Organisms/strains**		

Transgenic B6-Foxp3^EGFP^ mice (B6.Cg-Foxp3^tm2(EGFP)Tch^/J)	The Jackson Laboratory	006772

**Software and algorithms**		

GraphPad Prism	GraphPad	
FlowJo	Becton Dickinson	

### Experimental model and study participant details

#### Transgenic B6-Foxp3^EGFP^ mice

All experiments were conducted using male and female transgenic B6-Foxp3^EGFP^ mice (B6.Cg-*Foxp3*^*tm2(EGFP)Tch*^/J, The Jackson Laboratory, stock no. 006772), aged between 8 and 26 weeks. The animals were bred and housed in specific pathogen-free conditions at the Joint Animal Laboratory, part of Utrecht University. All procedures involving animals were approved by the Animal Welfare Body of Utrecht University and conducted in accordance with institutional guidelines and ethical standards. The research was carried out under the ethical license of the University Medical Center Utrecht, ensuring full compliance with the European Directive 2010/63/EU on the protection of animals used for scientific purposes.

#### Cord blood

Umbilical cord blood from healthy male and female donors was collected in accordance with the Declaration of Helsinki. The protocol for collection was reviewed and approved by the Ethics Committee of the University Medical Center Utrecht (UMCU), the Netherlands.

#### Cell lines

Daudi cells (Burkitt lymphoma, DSMZ ACC 78) were cultured in RPMI 1640 GlutaMAX HEPES culture medium (Life Technologies) supplemented with 10% fetal bovine serum (FBS, Sigma) and 100 μg/mL penicillin–streptomycin (Life Technologies). Cells were authenticated by fingerprinting (DNA – STR profiling, Eurofins), and tested negative for mycoplasma contamination.

### Method details

#### Rheological characterization by sweep tests

Rheology experiments on gel precursor solutions to determine the crosslinking kinetics were assessed using a DHR2 rheometer (TA Instruments, The Netherlands). For rheology assays, gel precursors were prepared at the same concentrations used for T cell culture as described below (Matrigel and BME were diluted 1:1 in cell culture medium, and 1.5% nanofibrillar cellulose hydrogel (GrowDex) was diluted to 0.25% w/v in culture medium). Time/temperature sweep experiments were performed at a frequency of 1.0 Hz, angular frequency of 6.283 rad/s, with 5.0% constant strain starting at 4°C with an increase of 5°C per minute until 37°C was reached, then the temperature kept constant for the duration of the experiment (n = 3 independent samples). Subsequently, frequency sweep experiments were performed at a frequency range from 0.1 Hz to 100 Hz with 5.0% constant strain at 37°C. Subsequently, amplitude sweep experiments were performed at a frequency of 1.0 Hz at a strain rate from 0.1% to 100% strain at 37°C. A volume of 100 μL of gel was used with a gap size of 300 μm. A 20.0 mm parallel EHP stainless steel plate was used as geometry.

#### Murine CD4^+^ T cell isolation

CD4^+^ T cells were isolated from (lymph nodes (inguinal, brachial, axillary, and cervical) and spleens of transgenic B6-Foxp3^EGFP^ mice. Tissues were mechanically dissociated by pressing through a cell strainer to obtain single-cell suspensions. Cells were centrifuged at 400 g for 4 minutes at 4°C and resuspended in MACS buffer (2% heat-inactivated FBS (Gemini Bio-Products) and 2 mM EDTA in PBS), followed by cell counting using a TC20 automated cell counter (Bio-Rad). CD4^+^ T cells were isolated using mouse CD4 (L3T4) microbeads (Miltenyi Biotec) according to the manufacturer’s protocol. Each mouse sample was processed with one LC column (Miltenyi Biotec) tightly placed in the QuadroMACS™ Separator (Miltenyi Biotec).

#### Human CD4^+^ T cell isolation

CD4^+^ T cells were isolated from cord blood of healthy donors Following density gradient centrifugation with Ficoll-Paque (GE Healthcare) gradient separation, cord blood mononuclear cells (CBMCs) were cryopreserved for later use. CD4^+^ T cells were subsequently enriched from thawed CBMCs using the MagniSort™ Human CD4^+^ T Cell Enrichment Kit (Thermo Fisher) in MACS buffer and the BD IMag™ Cell Separation Magnet (BD Biosciences), according to the manufacturer’s instructions.

#### Cell encapsulation in NFC, Matrigel and BME

Cell suspensions were spun down (1500 rpm, 5 minutes, 4°C) and pellets we re resuspended in either NFC (GrowDex, UPM Biomedicals), Matrigel (Corning) or BME (Cultrex® RGF BME Type 2, R&D systems). GrowDex (supplied as a suspension of 1.5% nanofibrillar cellulose in 98.5% ultra-pure water) was diluted to 0.25% in culture medium, using low-retention pipet tips. Cell pellets were resuspended in 70 mL of this 0.25% NFC suspension, and distributed in 96-well-U plates. 130 mL of culture medium supplemented with soluble IL-2 (20 U/mL, PeproTech) were carefully pipetted on top of the gel. For encapsulation in Matrigel and BME, micro centrifuge tubes containing the cell pellets were placed on ice and resuspended in 70 μL of cold Matrigel or BME diluted 1:1 in culture medium. Gel crosslinking was induced by incubating the gel precursors at 37 °C for 30 min. After this time, 130 μL of medium supplemented with soluble IL-2 (20 U/mL, PeproTech) was carefully pipetted on top of the gels. After culture, cells were recovered from NFC hydrogels by adding GrowDase (UPM Biomedicals) (800 mg GrowDase per mg of GrowDex) and incubating at 37 °C for 3 h. Matrigel and BME were disrupted by using Cell Recovery Solution (Corning). All samples were filtered through a 30 mM cell strainer before flow cytometric analysis.

#### Culture of murine and human CD4^+^ T cells

Murine and human CD4^+^ T cells were labelled with CellTrace Violet (Life technologies) and cultured (200.000 cells/well, 96-well-plate) in either a control 2D suspension (final volume/well: 200 μL), or the different hydrogels, as specified above. Human CD4^+^ T cells were cultured in RPMI10% medium (RPM1 1640 medium with Glutamax (Gibco) supplemented with 10% heat-inactivated FBS and 100 U/mL penicillin-streptomycin (Thermo Fisher Scientific). Murine CD4^+^ T cells were cultured in RPMI10% supplemented with 1 mM sodium pyruvate (Gibco), 1X nonessential amino acids (Gibco), 10 mM HEPES (Gibco) and 50 μM 2-mercaptoethanol (Sigma-Aldrich). Functional grade monoclonal antibodies directed against mouse or human CD3 (anti-CD3, 1 μg/ml) and CD28 (anti-CD28, 1 μg/ml) (eBioscience) were either incorporated into the matrices or added to the cell culture medium in the 2D controls. After 5 days in culture at 37°C, CD4^+^ T cells were retrieved from the gels as specified above and stained for flow cytometry analysis. For intracellular staining of IFNγ, GolgiStop (containing Monensin, BD Biosciences) was added to the medium 4 hours before staining. In the case of NFC gels, GrowDase (2 μg/ml, UPM Biomedicals) was added simultaneously with GolgiStop.

#### Flow cytometry analysis of CD4^+^ T cells

Murine and human CD4^+^ T cells were stained with live/dead dye Zombie NIR (BioLegend) in PBS, followed by staining with fluorochrome-labeled antibodies in MACS buffer. Murine CD4^+^ T cells were stained with anti-CD4-APC (BioLegend) and anti-CD25-Pacific blue (BioLegend). Human CD4^+^ T cells were stained with anti-CD4-FITC (BioLegend) and anti-CD25-APC (BioLegend). For the assessment of human Treg cell differentiation, CD4^+^ T cells underwent intracellular staining using the Foxp3/Transcription Factor Staining Buffer Set (eBioscience Thermofisher) with anti-FOXP3-PE (Biolegend). For both murine and human cells, intracellular IFNγ-staining was carried out using the Intracellular Fixation & Permeabilization Buffer Set (eBioscience Thermofisher) with anti- IFNγ-PECy7 (BD Biosciences for human and Biolegend for murine CD4^+^ T cells). Flow cytometry data were acquired using a BD LSRFortessa Cell Analyzer (BD Biosciences) with FACSDiva (BD Biosciences) software or a CytoFLEX Flow Cytometer (Beckman Coulter) with CytExpert software. Data were analyzed with FlowJo (Treestar, 10.6.2) or CytExpert software (2.4), respectively.

#### CD20 CAR-T cell generation and culture

The CD20 CAR construct (pBu-CD20–CAR) was generated by cloning single-chain variable fragments from anti-CD20 antibody rituximab into a pBullet vector containing a D8α−41BB-CD3-ζ signaling cassette. Phoenix-Ampho packaging cells were transfected with gag-pol (pHit60), env (P-COLT-GALV) and pBu-CD20–CAR using FugeneHD transfection reagent (Promega). Human peripheral blood mononuclear cells (PBMCs) were preactivated with 30 ng/mL anti-CD3 (OKT3, Miltenyi) and 50 IU/mL IL-2 (PeproTech) and subsequently transduced two times with viral supernatant in the presence of 6 μg/mL polybrene (Sigma) and 50 U/mL IL-2. Transduced T cells were expanded using 50 U/mL IL-2 and anti CD3/CD28 dynabeads (Thermo Fisher), and CD20–CAR-expressing cells were selected by treatment with 80 μg/mL neomycin. T cells were further expanded using rapid expansion protocol as described elsewhere.^56^

#### CD20 CAR-T cell mediated cytotoxicity assays

For cytotoxicity assays, Daudi cells were labelled with CellTrace Violet dye (Invitrogen) and mixed with CD20 CAR-T cells at the effector to target (E:T) ratios indicated (50.000 Daudi cells/well). Cells were spun down and resuspended in the different hydrogels as specified above. After 24 h, gels were digested and assessment of cell viability and activation was performed by staining with anti-CD69 FITC (Miltenyi) and 20 nM TO-PRO-3 (Thermo Scientific). Intracellular IFNγ-staining was carried out using the Intracellular Fixation & Permeabilization Buffer Set (eBioscience Thermofisher) with anti- IFNγ-PECy7 (BD Biosciences). The number of alive cells recovered from culture was quantified by flow cytometry, using Flow-count fluorospheres (Beckman Coulter).

#### CAR-T cell proliferation and phenotype analysis

CD20 CAR-T cells were labelled with CellTrace Violet (Life technologies) and cultured (50.000 cells/well) in either 2D or in hydrogels, as specified above. CAR-T cells were cultured in huRPMI2.5% medium (RPM1 1640 medium with GlutaMAX, supplemented with 2.5% pooled human serum, 100 μg/mL penicillin–streptomycin, 50 μM 2-mercaptoethanol and 50 IU/mL IL-2). Cells were stimulated with coated anti-CD3 (1.6 μg/mL) and soluble anti-CD28 (1 μg/mL) functional grade monoclonal antibodies (Miltenyi). After 4 or 10 days in culture at 37°C, CAR-T cells were recovered from the gels as described above. Cells were stained with anti-CD4 PECy7 (Invitrogen), anti-CD25 AF488 (Biolegend), anti-TIM-3 PE (Biolegend), anti-HLA-DR PECy7 (Biolegend) and 20 nM TO-PRO-3 to assess viability and activation. Samples were acquired in a LSRFortessa (BD Biosciences) with FACSDiva software and analyzed with FlowJo (Treestar, 10.6.2).

### Quantification and statistical analysis

Data were analyzed using GraphPad Prism 10. Mean values with corresponding standard deviations (SD) are presented, and a minimum of three experiments were conducted for each group (refer to Figure Legends for additional details). Statistical significance was determined through ordinary one-way ANOVA with Tukey’s multiple comparison test unless otherwise stated. Significance levels are denoted by asterisks: *P ≤* 0.05 (∗), *P ≤* 0.01 (∗∗), or *P ≤* 0.001 (∗∗∗) or *P* < 0.0001 (∗∗∗∗). Details specific to each figure are provided in the corresponding figure legends.
