# Supplementary material for: Impact of 3D cell culture hydrogels derived from basement membrane extracts or nanofibrillar cellulose on CAR-T cell activation
Source: iScience. 2025 Jul 30;28(9):113234. doi: 10.1016/j.isci.2025.113234 (PMC12362413; doi:10.1016/j.isci.2025.113234)
Supplement: Document S1. Figures S1 and S2 [file mmc1.pdf]

iScience, Volume *28*

Supplemental information

Impact of 3D cell culture hydrogels derived from basement membrane extracts or nanofibrillar cellulose on CAR-T cell activation

Sonia Aristin Revilla, Alessandro Cutilli, Eugenia Cambiaso, Dedeke Rockx-Brouwer, Cynthia Lisanne Frederiks, Marc Falandt, Riccardo Levato, Onno Kranenburg, Caroline A. Lindemans, Paul James Coffer, Victor Peperzak, Enric Mocholi, and Marta Cuenca

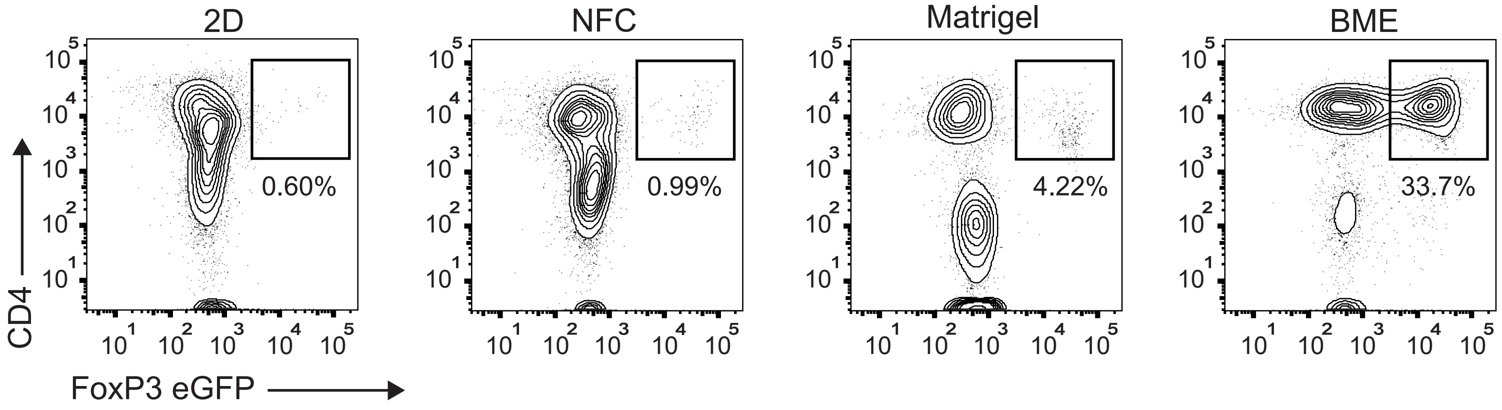

Supplemental Figure 1. Increased Treg cell percentage when CD4+ T cells are cultured in BME or Matrigel. CD4+ T cells were isolated from Foxp3eGFP mice and stimulated ex-vivo with anti-CD3 (1 µg/ml) and anti-CD28 (1 µg/ml) in 2D suspension (control) or embedded in different hydrogels (NFC, Matrigel and BME) for 5 days. Representative flow cytometry plots show the proportion of FoxP3 eGFP+CD4+ cells in each condition (gated on Alive cells). NFC, nanofibrillar cellulose; MG, Matrigel; BME, basement membrane extract.

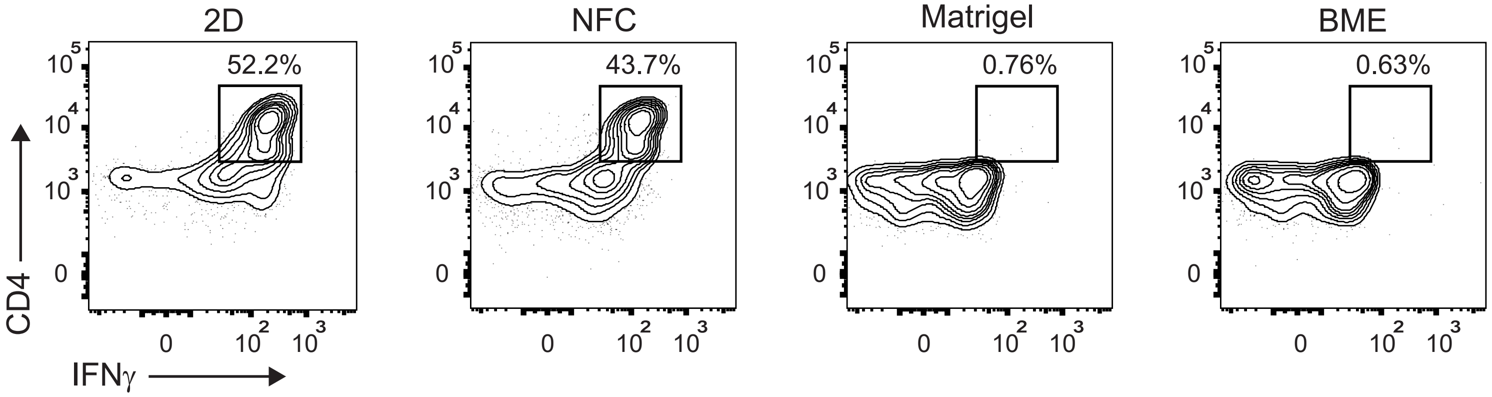

Supplemental Figure 2. Matrigel and BME hinder CD4+ T cell activation. CD4+ T cells were isolated from cord blood mononuclear cells and stimulated ex-vivo with anti-CD3 (1 µg/ml) and anti-CD28 (1 µg/ml) in 2D suspension (control) or embedded in differenthydrogels (NFC, Matrigel and BME) for 5 days. Representative flow cytometry plots show the proportion of IFNg+CD4+ cells in each condition (gated on Alive cells). NFC, nanofibrillar cellulose; MG, Matrigel; BME, basement membrane extract.
